# 4,4′-Dichloro-2,2′-[imidazolidine-1,3-diylbis(methylene)]diphenol

**DOI:** 10.1107/S1600536811035677

**Published:** 2011-09-14

**Authors:** Augusto Rivera, John Sadat-Bernal, Jaime Ríos-Motta, Michaela Pojarová, Michal Dušek

**Affiliations:** a Departamento de Química, Universidad Nacional de Colombia, Ciudad Universitaria, Bogotá, Colombia; b Institute of Physics, AS CR, v.v.i., Na Slovance 2, 182 21 Praha 8, Czech Republic

## Abstract

The imidazolidine ring in the title compound, C_17_H_18_Cl_2_N_2_O_2_, adopts a twist conformation. The observed conformation is stabilized by two intra­molecular O—H⋯N hydrogen bonds, with both N atoms acting as hydrogen-bond acceptors. The phenyl substituents are aligned at 70.0 (1) and 76.6 (1)° with respect to the best plane through the five atoms of the imidazolidine ring. Weak inter­molecular C—H⋯O inter­actions stabilize the crystal packing.

## Related literature

For the preparation of the title compound, see: Rivera *et al.* (1993 ▶). For synthetic applications of these di-Mannich bases, see: Rivera & Quevedo (2004 ▶); Rivera *et al.* (2004 ▶). For a closely related structure, see: Rivera *et al.* (2010 ▶). For puckering parameters, see: Cremer & Pople (1975 ▶). For applications of tetra­hydro­salens and heterocalixarenes in medicine and metal-complex catalysis, see: Balsells & Walsh (2000 ▶); Weber *et al.* (1996 ▶).
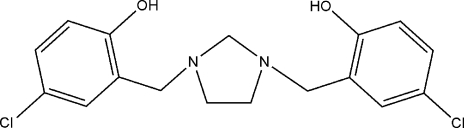


## Experimental

### 

#### Crystal data

C_17_H_18_Cl_2_N_2_O_2_
*M* *_r_* = 353.23
Monoclinic, 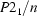

*a* = 10.8640 (2) Å
*b* = 9.6125 (2) Å
*c* = 16.7242 (4) Å
β = 106.608 (2)°
*V* = 1673.65 (6) Å^3^
*Z* = 4
Cu *K*α radiation
μ = 3.58 mm^−1^
*T* = 120 K
0.42 × 0.37 × 0.25 mm

#### Data collection

Oxford Diffraction Xcalibur Atlas Gemini ultra diffractometer
Absorption correction: analytical (*CrysAlis PRO*; Oxford Diffraction, 2010 ▶) *T*_min_ = 0.669, *T*_max_ = 0.777
19547 measured reflections
2994 independent reflections
2772 reflections with *I* > 2σ(*I*)’
*R*_int_ = 0.040

#### Refinement

*R*[*F* ^2^ > 2σ(*F* ^2^)] = 0.035
*wR*(*F* ^2^) = 0.101
*S* = 1.04
2994 reflections
208 parameters
H-atom parameters constrained
Δρ_max_ = 0.29 e Å^−3^
Δρ_min_ = −0.30 e Å^−3^

### 

Data collection: *CrysAlis PRO* (Oxford Diffraction, 2010 ▶); cell refinement: *CrysAlis PRO*; data reduction: *CrysAlis PRO*; program(s) used to solve structure: *SHELXS97* (Sheldrick, 2008 ▶); program(s) used to refine structure: *SHELXL97* (Sheldrick, 2008 ▶); molecular graphics: *DIAMOND* (Brandenburg & Putz, 2005 ▶); software used to prepare material for publication: *publCIF* (Westrip, 2010 ▶).

## Supplementary Material

Crystal structure: contains datablock(s) I, global. DOI: 10.1107/S1600536811035677/bt5631sup1.cif

Structure factors: contains datablock(s) I. DOI: 10.1107/S1600536811035677/bt5631Isup2.hkl

Supplementary material file. DOI: 10.1107/S1600536811035677/bt5631Isup3.cml

Additional supplementary materials:  crystallographic information; 3D view; checkCIF report

## Figures and Tables

**Table 1:** Hydrogen-bond geometry (Å, °)

*D*—H⋯*A*	*D*—H	H⋯*A*	*D*⋯*A*	*D*—H⋯*A*
O1—H1*O*1⋯N1	0.97	1.77	2.6524 (17)	149
O2—H1*O*2⋯N2	0.96	1.77	2.6515 (17)	150
C4—H4*B*⋯O2^i^	0.97	2.52	3.466 (2)	163
C9—H9⋯O2^ii^	0.93	2.47	3.395 (2)	172
C11—H11*B*⋯O1^iii^	0.97	2.58	3.482 (2)	154

Symmetry codes: (i) 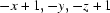; (ii) 
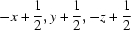
; (iii) 
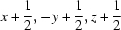
.

**Fig. 1.**
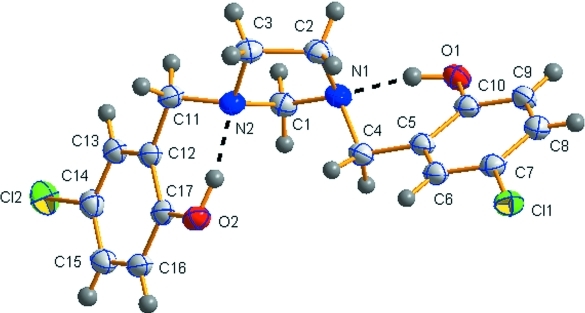
A view of (I) with the numbering scheme. Displacement ellipsoids are drawn at the 50% probability level.

**Fig. 2.**
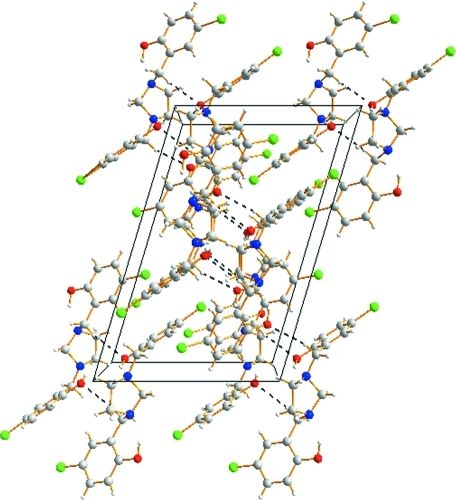
Packing of the molecules of the title compound view along b axis.

## supplementary crystallographic information

### Comment

In connection with our synthetic studies on heterocyclic compounds we earlier synthesized a series of *di-*Mannich bases named 2,2'-(imidazolidine-1,3-diyldimethanediyl)bis(4-substitutedphenol) by reaction of appropriate phenols with 1,3,6,8-tetraazatricyclo[4.4.1.1^3,8^]dodecane (Rivera *et al.*, 1993). They are promising synthetic intermediates for the synthesis of tetrahydrosalens (Rivera, Quevedo, Navarro & Maldonado, 2004) and heterocalixarenes (Rivera & Quevedo, 2004), which find wide use in both medicine and metal-complex catalysis (Balsells & Walsh, 2000; Weber *et al.* 1996). These Mannich bases are convenient models for studying the nature of hydrogen bonding and weak noncovalent interactions, which play a key role in biological processes and design of complex structures.

We report here the structure of the title compound (I) (Fig. 1), which was prepared according to the previously reported procedure (Rivera *et al.*, 1993) but using the intriguing aminal 1,3,6,8-tetraazatricyclo[4.3.1.1^3,8^]undecane. Recrystallization from methanol by slow evaporation over a period of one week affording crystals suitable for X-ray analysis.

The asymmetric unit of (I), Fig 1, contains one independent 2,2'-(imidazolidine-1,3-diylbis(methylene))bis(4-chlorophenol) molecule. Distances and angles are similar to those observed before in the closely related structure 4,4'-dichloro-2,2'-[(3aR,7aR/3aS,7aS)-2,3,3a,4,5,6,7,7a-octahydro-*1H*-1,3-benzimidazole-1,3-diyl)bis(methylene)]diphenol (Rivera *et al.*, 2010). The imidazolidine ring is in a twist conformation on C1–N2 with Q(2) 0.402 (2) Å and φ 52.7 (2)° (Cremer & Pople, 1975). Its central ring makes an angle of 70.0 (1)° and 76.6 (1)° with the planar phenyl rings (C5—C10) and (C12—C17) respectively. The crystal structure has two intramolecular hydrogen bonds and three C—H···O intermolecular hydrogen bonds (Table 1). The unit cell contains four molecules of the title compound (I), which form pairs of hydrogen bonded dimers (Table 1, Figs. 2). Neighboring pairs of these dimers are orthogonally arranged with respect to each other. Lattice binding is provided principally by C—H···O interactions, shown in Figure 2. The chains, aligned along the *c* axis, are further linked together *via* cross-linking weaker C—H···O interactions (Table 1).

### Experimental

For the originally reported synthesis, see: Rivera *et al.* (1993)

### Refinement

All H atoms could be located in a difference Fourier synthesis. Nevertheless, H atoms were refined as riding with H bonded to O at the positions where they were found and C–H distances of 0.93 Å for aromatic H and C–H = 0.97Å for methylene groups. All H atoms were refined with   displacement coefficients *U*_iso_(H) set to *1.5U*_eq_(O) for hydroxyl groups and to *1.2U*_eq_(C) for the CH– and CH_2_– groups.

### Crystal data

**Table:**

C_17_H_18_Cl_2_N_2_O_2_	*F*(000) = 736
*M**_r_* = 353.23	*D*_x_ = 1.402 Mg m^−^^3^
Monoclinic, *P*2_1_/*n*	Cu *K*α radiation, λ = 1.5418 Å
Hall symbol: -P 2yn	Cell parameters from 9456 reflections
*a* = 10.8640 (2) Å	θ = 4.2–67.1°
*b* = 9.6125 (2) Å	µ = 3.58 mm^−^^1^
*c* = 16.7242 (4) Å	*T* = 120 K
β = 106.608 (2)°	Prism, colourless
*V* = 1673.65 (6) Å^3^	0.42 × 0.37 × 0.25 mm
*Z* = 4	

### Data collection

**Table:**

Oxford Diffraction Xcalibur Atlas Gemini ultra diffractometer	2994 independent reflections
Radiation source: Enhance Ultra (Cu) X-ray Source	2772 reflections with *I* > 2σ(*I*)'
mirror	*R*_int_ = 0.040
Detector resolution: 10.3784 pixels mm^-1^	θ_max_ = 67.2°, θ_min_ = 4.4°
Rotation method data acquisition using ω scans	*h* = −12→12
Absorption correction: analytical (*CrysAlis PRO*; Oxford Diffraction, 2010)	*k* = −11→11
*T*_min_ = 0.669, *T*_max_ = 0.777	*l* = −19→17
19547 measured reflections	

### Refinement

**Table:**

Refinement on *F*^2^	Primary atom site location: structure-invariant direct methods
Least-squares matrix: full	Secondary atom site location: difference Fourier map
*R*[*F*^2^ > 2σ(*F*^2^)] = 0.035	Hydrogen site location: inferred from neighbouring sites
*wR*(*F*^2^) = 0.101	H-atom parameters constrained
*S* = 1.04	*w* = 1/[σ^2^(*F*_o_^2^) + (0.0604*P*)^2^ + 0.5161*P*] where *P* = (*F*_o_^2^ + 2*F*_c_^2^)/3
2994 reflections	(Δ/σ)_max_ < 0.001
208 parameters	Δρ_max_ = 0.29 e Å^−^^3^
0 restraints	Δρ_min_ = −0.30 e Å^−^^3^

### Special details

**Table:**

Geometry. All e.s.d.'s (except the e.s.d. in the dihedral angle between two l.s. planes) are estimated using the full covariance matrix. The cell e.s.d.'s are taken into account individually in the estimation of e.s.d.'s in distances, angles and torsion angles; correlations between e.s.d.'s in cell parameters are only used when they are defined by crystal symmetry. An approximate (isotropic) treatment of cell e.s.d.'s is used for estimating e.s.d.'s involving l.s. planes.
Refinement. Refinement of *F*^2^ against ALL reflections. The weighted *R*-factor *wR* and goodness of fit *S* are based on *F*^2^, conventional *R*-factors *R* are based on *F*, with *F* set to zero for negative *F*^2^. The threshold expression of *F*^2^ > σ(*F*^2^) is used only for calculating *R*-factors(gt) *etc*. and is not relevant to the choice of reflections for refinement. *R*-factors based on *F*^2^ are statistically about twice as large as those based on *F*, and *R*- factors based on ALL data will be even larger. The H atoms were all located in a difference map, but those attached to carbon atoms were repositioned geometrically. The isotropic temperature parameters of hydrogen atoms were calculated as 1.2**U*_eq_ of the parent atom. The distance between hydrogen and oxygen atom in hydroxyl group was fixed to the distance 0.87 Å.

### Fractional atomic coordinates and isotropic or equivalent isotropic displacement parameters (Å^2^)

**Table:**

	*x*	*y*	*z*	*U*_iso_*/*U*_eq_	
C1	0.21837 (14)	0.21659 (16)	0.42784 (10)	0.0322 (3)	
H1A	0.1749	0.1280	0.4260	0.039*	
H1B	0.1608	0.2905	0.4337	0.039*	
C2	0.36940 (16)	0.33596 (16)	0.38001 (10)	0.0368 (4)	
H2A	0.3437	0.4280	0.3576	0.044*	
H2B	0.4409	0.3050	0.3607	0.044*	
C3	0.40707 (16)	0.33848 (17)	0.47479 (10)	0.0372 (4)	
H3A	0.4991	0.3275	0.4981	0.045*	
H3B	0.3809	0.4250	0.4949	0.045*	
C4	0.29989 (15)	0.10433 (15)	0.32188 (10)	0.0320 (3)	
H4A	0.2325	0.0354	0.3154	0.038*	
H4B	0.3766	0.0692	0.3621	0.038*	
C5	0.32658 (14)	0.12712 (15)	0.23931 (9)	0.0303 (3)	
C6	0.42918 (14)	0.06128 (15)	0.22089 (9)	0.0312 (3)	
H6	0.4843	0.0051	0.2607	0.037*	
C7	0.44951 (15)	0.07915 (16)	0.14360 (10)	0.0326 (3)	
C8	0.37107 (16)	0.16444 (16)	0.08356 (10)	0.0354 (4)	
H8	0.3864	0.1763	0.0320	0.043*	
C9	0.26963 (16)	0.23171 (16)	0.10149 (10)	0.0351 (4)	
H9	0.2168	0.2901	0.0619	0.042*	
C10	0.24590 (14)	0.21281 (15)	0.17805 (10)	0.0316 (3)	
C11	0.31970 (15)	0.23442 (16)	0.58004 (10)	0.0330 (3)	
H11A	0.2605	0.3103	0.5793	0.040*	
H11B	0.4013	0.2578	0.6198	0.040*	
C12	0.26818 (14)	0.10368 (16)	0.60816 (9)	0.0299 (3)	
C13	0.18268 (15)	0.11162 (16)	0.65594 (9)	0.0328 (3)	
H13	0.1516	0.1976	0.6667	0.039*	
C14	0.14386 (17)	−0.00807 (17)	0.68735 (11)	0.0358 (4)	
C15	0.18658 (17)	−0.13748 (17)	0.67129 (10)	0.0387 (4)	
H15	0.1597	−0.2173	0.6929	0.046*	
C16	0.26995 (16)	−0.14685 (17)	0.62260 (10)	0.0361 (4)	
H16	0.2985	−0.2336	0.6109	0.043*	
C17	0.31142 (15)	−0.02753 (16)	0.59103 (10)	0.0311 (3)	
N1	0.25961 (12)	0.23659 (13)	0.35315 (8)	0.0322 (3)	
N2	0.33788 (12)	0.21975 (13)	0.49679 (8)	0.0300 (3)	
O1	0.14312 (10)	0.27831 (11)	0.19253 (7)	0.0364 (3)	
H1O1	0.1592	0.2802	0.2528	0.044*	
O2	0.39647 (10)	−0.04067 (12)	0.54511 (7)	0.0348 (3)	
H1O2	0.4004	0.0494	0.5208	0.042*	
Cl1	0.57874 (4)	−0.00643 (4)	0.12223 (3)	0.03906 (15)	
Cl2	0.04238 (5)	0.00586 (5)	0.75129 (3)	0.04919 (16)	

### Atomic displacement parameters (Å^2^)

**Table:**

	*U*^11^	*U*^22^	*U*^33^	*U*^12^	*U*^13^	*U*^23^
C1	0.0287 (7)	0.0310 (8)	0.0365 (8)	0.0010 (6)	0.0086 (6)	0.0027 (6)
C2	0.0402 (9)	0.0281 (8)	0.0418 (9)	−0.0043 (6)	0.0116 (7)	0.0039 (6)
C3	0.0384 (8)	0.0313 (8)	0.0417 (9)	−0.0080 (6)	0.0111 (7)	−0.0005 (6)
C4	0.0335 (8)	0.0265 (7)	0.0346 (8)	0.0011 (6)	0.0073 (6)	0.0047 (6)
C5	0.0323 (7)	0.0237 (7)	0.0317 (8)	−0.0034 (6)	0.0039 (6)	0.0025 (6)
C6	0.0316 (7)	0.0245 (7)	0.0332 (8)	−0.0015 (6)	0.0025 (6)	0.0014 (6)
C7	0.0343 (8)	0.0257 (7)	0.0368 (8)	−0.0026 (6)	0.0083 (6)	−0.0026 (6)
C8	0.0437 (9)	0.0294 (8)	0.0321 (8)	−0.0033 (6)	0.0090 (7)	0.0008 (6)
C9	0.0404 (9)	0.0273 (8)	0.0328 (8)	0.0009 (6)	0.0025 (7)	0.0046 (6)
C10	0.0312 (7)	0.0240 (7)	0.0360 (8)	−0.0020 (6)	0.0043 (6)	0.0008 (6)
C11	0.0367 (8)	0.0263 (7)	0.0355 (8)	−0.0008 (6)	0.0094 (7)	−0.0034 (6)
C12	0.0289 (7)	0.0286 (8)	0.0290 (7)	0.0006 (6)	0.0029 (6)	−0.0009 (6)
C13	0.0337 (8)	0.0322 (8)	0.0301 (8)	0.0025 (6)	0.0052 (6)	0.0002 (6)
C14	0.0339 (8)	0.0402 (9)	0.0307 (8)	−0.0035 (6)	0.0053 (7)	0.0011 (6)
C15	0.0442 (9)	0.0320 (8)	0.0350 (8)	−0.0078 (7)	0.0036 (7)	0.0040 (7)
C16	0.0415 (9)	0.0266 (7)	0.0351 (8)	0.0008 (6)	0.0026 (7)	−0.0015 (6)
C17	0.0299 (7)	0.0296 (7)	0.0291 (8)	0.0007 (6)	0.0008 (6)	−0.0018 (6)
N1	0.0327 (6)	0.0264 (6)	0.0368 (7)	0.0001 (5)	0.0089 (6)	0.0035 (5)
N2	0.0286 (6)	0.0279 (6)	0.0330 (7)	−0.0015 (5)	0.0081 (5)	−0.0007 (5)
O1	0.0341 (6)	0.0341 (6)	0.0385 (6)	0.0057 (4)	0.0063 (5)	0.0060 (5)
O2	0.0353 (6)	0.0299 (5)	0.0395 (6)	0.0045 (4)	0.0109 (5)	−0.0021 (5)
Cl1	0.0404 (3)	0.0345 (2)	0.0437 (3)	0.00186 (14)	0.01431 (19)	−0.00169 (15)
Cl2	0.0477 (3)	0.0585 (3)	0.0470 (3)	−0.00269 (18)	0.0226 (2)	0.00619 (18)

### Geometric parameters (Å, °)

**Table:**

C1—N1	1.455 (2)	C8—H8	0.9300
C1—N2	1.470 (2)	C9—C10	1.388 (2)
C1—H1A	0.9700	C9—H9	0.9300
C1—H1B	0.9700	C10—O1	1.3625 (19)
C2—N1	1.494 (2)	C11—N2	1.468 (2)
C2—C3	1.520 (2)	C11—C12	1.505 (2)
C2—H2A	0.9700	C11—H11A	0.9700
C2—H2B	0.9700	C11—H11B	0.9700
C3—N2	1.470 (2)	C12—C13	1.390 (2)
C3—H3A	0.9700	C12—C17	1.404 (2)
C3—H3B	0.9700	C13—C14	1.380 (2)
C4—N1	1.4873 (19)	C13—H13	0.9300
C4—C5	1.506 (2)	C14—C15	1.381 (2)
C4—H4A	0.9700	C14—Cl2	1.7466 (18)
C4—H4B	0.9700	C15—C16	1.383 (3)
C5—C6	1.391 (2)	C15—H15	0.9300
C5—C10	1.408 (2)	C16—C17	1.390 (2)
C6—C7	1.383 (2)	C16—H16	0.9300
C6—H6	0.9300	C17—O2	1.366 (2)
C7—C8	1.385 (2)	O1—H1O1	0.9725
C7—Cl1	1.7500 (16)	O2—H1O2	0.9623
C8—C9	1.383 (2)		
			
N1—C1—N2	104.54 (12)	C8—C9—H9	119.7
N1—C1—H1A	110.8	C10—C9—H9	119.7
N2—C1—H1A	110.8	O1—C10—C9	118.76 (14)
N1—C1—H1B	110.8	O1—C10—C5	120.82 (14)
N2—C1—H1B	110.8	C9—C10—C5	120.42 (14)
H1A—C1—H1B	108.9	N2—C11—C12	112.28 (12)
N1—C2—C3	106.13 (12)	N2—C11—H11A	109.1
N1—C2—H2A	110.5	C12—C11—H11A	109.1
C3—C2—H2A	110.5	N2—C11—H11B	109.1
N1—C2—H2B	110.5	C12—C11—H11B	109.1
C3—C2—H2B	110.5	H11A—C11—H11B	107.9
H2A—C2—H2B	108.7	C13—C12—C17	118.97 (14)
N2—C3—C2	104.13 (12)	C13—C12—C11	120.21 (14)
N2—C3—H3A	110.9	C17—C12—C11	120.70 (14)
C2—C3—H3A	110.9	C14—C13—C12	120.02 (15)
N2—C3—H3B	110.9	C14—C13—H13	120.0
C2—C3—H3B	110.9	C12—C13—H13	120.0
H3A—C3—H3B	108.9	C13—C14—C15	121.39 (16)
N1—C4—C5	110.56 (12)	C13—C14—Cl2	118.96 (13)
N1—C4—H4A	109.5	C15—C14—Cl2	119.62 (13)
C5—C4—H4A	109.5	C14—C15—C16	119.09 (15)
N1—C4—H4B	109.5	C14—C15—H15	120.5
C5—C4—H4B	109.5	C16—C15—H15	120.5
H4A—C4—H4B	108.1	C15—C16—C17	120.51 (15)
C6—C5—C10	118.47 (14)	C15—C16—H16	119.7
C6—C5—C4	120.89 (13)	C17—C16—H16	119.7
C10—C5—C4	120.61 (14)	O2—C17—C16	118.82 (14)
C7—C6—C5	120.18 (14)	O2—C17—C12	121.17 (14)
C7—C6—H6	119.9	C16—C17—C12	120.00 (15)
C5—C6—H6	119.9	C1—N1—C4	112.49 (12)
C6—C7—C8	121.45 (14)	C1—N1—C2	103.98 (12)
C6—C7—Cl1	118.97 (12)	C4—N1—C2	111.25 (12)
C8—C7—Cl1	119.57 (12)	C11—N2—C1	114.70 (12)
C9—C8—C7	118.89 (15)	C11—N2—C3	112.33 (12)
C9—C8—H8	120.6	C1—N2—C3	102.70 (12)
C7—C8—H8	120.6	C10—O1—H1O1	106.2
C8—C9—C10	120.56 (14)	C17—O2—H1O2	105.8
			
?—?—?—?	?		

### Hydrogen-bond geometry (Å, °)

**Table:**

*D*—H···*A*	*D*—H	H···*A*	*D*···*A*	*D*—H···*A*
O1—H1O1···N1	0.97	1.77	2.6524 (17)	149
O2—H1O2···N2	0.96	1.77	2.6515 (17)	150
C4—H4B···O2^i^	0.97	2.52	3.466 (2)	163
C9—H9···O2^ii^	0.93	2.47	3.395 (2)	172
C11—H11B···O1^iii^	0.97	2.58	3.482 (2)	154

Symmetry codes: (i) −*x*+1, −*y*, −*z*+1; (ii) −*x*+1/2, *y*+1/2, −*z*+1/2; (iii) *x*+1/2, −*y*+1/2, *z*+1/2.
